# The Effect of Nicotinamide on Gene Expression in a Traumatic Brain Injury Model

**DOI:** 10.3389/fnins.2013.00021

**Published:** 2013-02-26

**Authors:** G. D. Anderson, T. C. Peterson, F. M. Farin, T. K. Bammler, R. P. Beyer, E. D. Kantor, M. R. Hoane

**Affiliations:** ^1^Department of Pharmacy, University of WashingtonSeattle, WA, USA; ^2^Department of Psychology, Southern Illinois UniversityCarbondale, IL, USA; ^3^Department of Environmental and Occupational Health Sciences, University of WashingtonSeattle, WA, USA

**Keywords:** nicotinamide, gene expression, cortical contusion model, traumatic brain injury

## Abstract

Microarray-based transcriptional profiling was used to determine the effect of nicotinamide on gene expression in an experimental traumatic brain injury (TBI) model. Ingenuity Pathway Analysis (IPA) was used to evaluate the effect on relevant functional categories and canonical pathways. At 24 h, 72 h, and 7 days, respectively, 70, 58, and 76%, of the differentially expressed genes were up-regulated in the vehicle treated compared to the sham animals. At 24 h post-TBI, there were 150 differentially expressed genes in the nicotinamide treated animals compared to vehicle; the majority (82%) down-regulated. IPA analysis identified a significant effect of nicotinamide on the functional categories of cellular movement, cell-to-cell-signaling, antigen presentation and cellular compromise, function, and maintenance and cell death. The canonical pathways identified were signaling pathways primarily involved with the inflammatory process. At 72 h post-cortical contusion injury, there were 119 differentially expressed genes in the nicotinamide treated animals compared to vehicle; the majority (90%) was up-regulated. IPA analysis identified a significant effect of nicotinamide on cell signaling pathways involving neurotransmitters, neuropeptides, growth factors, and ion channels with little to no effect on inflammatory pathways. At 7 days post-TBI, there were only five differentially expressed genes with nicotinamide treatment compared to vehicle. Overall, the effect of nicotinamide on counteracting the effect of TBI resulted in significantly decreased number of genes differentially expressed by TBI. In conclusion, the mechanism of the effect of nicotinamide on secondary injury pathways involves effects on inflammatory response, signaling pathways, and cell death.

## Introduction

The Centers for Disease Control and Prevention have stated that traumatic brain injury (TBI) is among the leading causes of acute and chronic disability in the United States and each year 1.7 million Americans endure a TBI, and 52,000 die (Faul et al., 2010). Although more individuals survive TBI than in the past, the survivors endure residual physical, cognitive, emotional, and/or behavioral impairments from the cascade of central pathological responses resulting from TBI.

The field of TBI recognizes two distinct forms or stages of brain injury. The first or primary injury relates to the initial injury caused by direct damage to the brain. It is believed that only injury prevention or reduction will reduce the consequences of the primary injury. The second is indirect and progressive and is referred to as secondary injury.

The etiology of secondary brain injury is multi-factorial, with a host of likely inter-related processes including mitochondrial energy failure, excessive generation of reactive oxygen species, activation of destructive enzymes such as poly (ADP-ribose) polymerase (PARP) and caspase family of proteases, membrane disruption, neuronal death, thrombosis due to intravascular coagulation in small vessels, increased synaptic concentrations of excitatory amino acids, and activation of innate inflammatory responses (Schouten, 2007). To date, no single pharmacological agent has been shown to improve the outcome of TBI. Several reviews have suggested the need for pharmacological treatments that target multiple secondary factors or combination treatment strategies (Faden and Stoica, 2007; Schouten, 2007).

Nicotinamide has been shown to improve neurological outcome and reduce the amount of tissue damage in various animal models TBI (Hoane et al., 2003, 2006a,b,c, 2008a,b; Holland et al., 2008; Goffus et al., 2010; Vonder Haar et al., 2011) and stroke (Ayoub et al., 1999; Sakakibara et al., 2002; Yang et al., 2002a,b,c). Recovery of function of both sensory and motor tasks after TBI have been found with a variety of nicotinamide dosing regiments which include doses ranging from 50 mg/kg to 500 mg/day kg administered up to 24 h post-injury to sustained infusion studies using 150 mg/day for 7 days.

Nicotinamide is the precursor of β-nicotinamide adenine dinucleotide (NAD+) and is important for the synthesis of nicotinamide adenine dinucleotide phosphate (NADP+). NAD+ and NADP+ are coenzymes that are vital to many oxidation-reduction reactions in cell metabolism. NAD+ is a precursor for ATP, and NAD+ increases neuronal ATP concentration (Klaidman et al., 1996). After TBI, nicotinamide may prevent the depletion of NAD+, thus protecting against ATP depletion and increasing neuroprotection (Li et al., 2006). Previous experimental studies have demonstrated that nicotinamide has anti-inflammatory and anti-oxidant effects, prevents apoptosis, and decreases cerebral edema (for reviews, see Yang and Adams, 2004; Li et al., 2006). However, the predominance of the preclinical studies evaluating the proposed mechanism of actions of nicotinamide have evaluated single or multiple 500 mg/kg doses, which result in peak concentrations of ∼300 μg/mL (Hoane et al., 2006c), 10–30 times higher than clinically relevant concentrations. Nicotinamide doses of 1–3 g/day (15–45 mg/kg/day; Knip et al., 2000) have been administered in the experimental treatment or prevention of diabetes type 2 and result in peak nicotinamide serum concentrations of ∼25–70 μg/mL (Horsman et al., 1993).

The objective of this study was to use microarray analysis to determine the effect of clinically relevant doses of nicotinamide on gene expression patterns and profiles following experimental TBI in the rat. By understanding the mechanism of the effect of nicotinamide on the secondary injury response in TBI, we can develop future treatment strategies using a combination of drugs to target the multiple pathways involved in secondary injury.

## Materials and Methods

### Animals

Male, Sprague-Dawley rats (Harlan, Indianapolis, IN, USA), 4-months of age were used in this study. All animal and surgical procedures were adhered to as described in the NIH Guide for the Care and Use of Laboratory Animals. The Southern Illinois University Institutional Animal Care and Use Committee (IACUC) and the University of Washington’s IACUC reviewed and approved all experimental procedures. Before and after injury, animals were housed in the university-maintained vivarium, with a 12-h light/dark schedule and a controlled environmental temperature of 22°C in standard housing cages with food and water available *ad libitum*.

### Pharmacokinetic studies

Male Sprague-Dawley rats (309 ± 17 g) with surgically implanted jugular vein catheters were obtained from Harlan Laboratories. Four animals were anesthetized using a mixture of isoflurane (2–4%) and oxygen (0.8 L/min). Alzet osmotic mini-pumps (Alzet model 2ML1, pumping rate of 10 μL/h) were implanted subcutaneously (s.c.) ∼2.0 cm posterior to the scapulae and shifted to the right of midline. The infusion pumps were weighed prior to and after loading the nicotinamide solution. After filling, the pumps were soaked overnight in a water bath at 37°C, and taken out just prior to placing in rat to insure that the pump was operating at the desired flow rate at the time of implantation. Based on the pharmacokinetic results of a previous single dose study (data not shown), a loading dose of 75 mg/kg of nicotinamide (Sigma-Aldrich Co., St. Louis, MO, USA) dissolved in phosphate buffered saline (PBS) was administered by s.c. injection and an infusion rate of 12.0 mg/h kg were used to target a steady state concentration of ∼50 μg/mL. Blood samples were collected in microtubes immediately prior to loading dose, and at 1, 4, 24, 48, and 72 h, and stored at −80°C until assayed using a previously published assay (Goffus et al., 2010).

### Experimental injury model

The cortical contusion injury (CCI) model utilized in the present study was based on previous studies and was intended to produce a moderately severe injury (Quigley et al., 2009; Goffus et al., 2010; Anderson et al., 2011). Animals, ∼3 months of age with a weight of 335 ± 28 g were anesthetized using a mixture of isoflurane (2–4%) and oxygen (0.8 L/min). When the animal became unresponsive (no ocular or pedal reflexes) the head was shaved and scrubbed with 70% alcohol followed by betadine and placed into a stereotaxic device. A midline incision was made in the skin as well as through the underlying fascia. A circular craniotomy (5.0 mm) was centered 2.4 mm posterior to and 2.4 mm lateral (left) to bregma. The contusion injury was created with the Benchmark™ stereotaxic impactor with a 4.0-mm diameter impactor tip (St. Louis, MO, USA)^1^. A moderate injury was induced with an impact speed of 3.0 m/s and an impact depth of 2.5 mm (Swan et al., 2011). The impact tip maintained contact with the brain tissue for 0.5 s before retraction. Normal body temperature (37°C) during surgery and recovery was maintained with a warm water recycling bed and pump system (EZ Anesthesia, Palmer, PA, USA). Four hours post-CCI, osmotic mini-pumps were implanted as described above. Rats receiving sham surgeries underwent identical surgical preparation as the injured animals but did not receive craniotomies or injuries, and were then sutured and allowed to recover.

Based on the pharmacokinetic studies, each animal received a 75-mg/kg s.c. loading dose of nicotinamide or vehicle (1.0 mL/kg PBS) at time of implantation of the infusion pump 4 h after the CCI injury. The nicotinamide infusion rate was 12 mg/h kg or vehicle (0.9% PBS) for 72 h post-injury as described above. Animals were randomly assigned to one of three groups: Group 1: Intact sham (*n* = 5), Group 2. CCI injured nicotinamide (*n* = 15), and Group 3. CCI injured vehicle (*n* = 15).

### Tissue harvest

**Figure 1:**
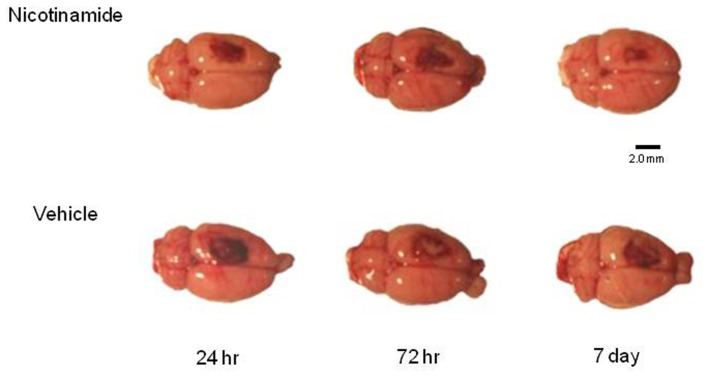
**Histology plate**. Shown are representative dorsal images of the injury in each group prior to the harvesting of the tissue for analysis. Image bar is 2.0 mm.

Five intact sham animals and five animals in each treatment group at specified time point’s post-CCI (24 h, 72 h, and 7 days) were overdosed with a mixture of CO_2_ (80%) and O_2_ (20%). The rats were then decapitated; a cardiac blood sample collected and brains were rapidly extracted. For the animals in the 7-day group, the infusion pump was explanted under anesthesia at 72 h post-TBI and a tail snip blood sample was obtained. To maintain quality control and to assure that all of the brains were injured, each brain was assigned a rating score (1 = no visual sign of trauma; 2 = bruised and swollen cortex; 3 = no remaining cortex; or extensive damage). Only brains with a score of 2 were used in the subsequent analyses. An example of representative dorsal images of the injury in each group prior to the harvesting of the tissue for analysis is shown in Figure 1.

The brain was then cut into a 4.0-mm coronal slab containing the injury site in a brain matrix (Braintree Scientific, Inc., Braintree, MA, USA) and was placed onto an RNAase free cold plate and a 5.0-mm biopsy bunch was used to collect the injury site and surrounding cortical tissue (Hoane et al., 2006a; Anderson et al., 2011). The tissue punch included all injured cortical tissue and a small strip of pericontusional tissue, with the ventral extent of the punch extending to the corpus callosum. Tissue punches were placed into microcentrifuge tubes, snap frozen, and then stored at −80°C. All samples were shipped by overnight carrier to the University of Washington on dry ice.

### Processing of samples for microarray analysis

Integrity of RNA samples was assessed with an Agilent 2100 Bioanalyzer (Agilent Technologies, Inc., Santa Clara, CA, USA) which is the method of choice and the recognized standard in the field. RNA integrity was judged by observing distinct and sharp 18 and 28 s ribosomal RNA peaks that were baseline separated. RNA quantity was determined by measuring OD_260_ with a Thermo Scientific NanoDrop™ 1000 Spectrophotometer (Thermo Fisher Scientific, Inc., Wilmington, DE, USA). The NanoDrop instrument was also used to determine purity of RNA samples by measuring OD_260/280_ and OD_260/230_ ratios. Only samples passing this stringent quality control were processed further. Processing of the RNA samples was carried out according to the Affymetrix GeneChip Whole Transcript Sense Target labeling protocol. Briefly, double-stranded cDNA was synthesized with random hexamers tagged with a T7 promoter sequence. The double-stranded cDNA was subsequently used as a template and amplified by T7 RNA polymerase producing many copies of antisense cRNA. In the second cycle of cDNA synthesis, random hexamers were used to prune reverse transcription of the cRNA from the first cycle to produce single-stranded DNA in the sense orientation. In order to reproducibly fragment the single-stranded DNA and improve the robustness of the assay, dUTP was incorporated in the DNA during the second cycle, first-strand reverse transcription reaction. This single-stranded DNA sample was then treated with a combination of uracil DNA glycosylase (UDG) and apurinic/apyrimidinic endonuclease 1 (APE 1) that specifically recognizes the unnatural dUTP residues and breaks the DNA strand. DNA was labeled by terminal deoxynucleotidyltransferase (TdT) with the Affymetrix^®^ proprietary DNA Labeling Reagent that is covalently linked to biotin. The biotin labeled DNA fragments were hybridized to Affymetrix GeneChip Rat Gene 1.0 ST arrays, washed, and stained with fluorescent anti streptavidin biotinylated antibody. Following an additional wash step, the arrays were scanned with an Affymetrix GeneChip^®^ 3000 scanner. Image generation and feature extraction was performed using Affymetrix GeneChip Command Console Software.

### Analysis of the microarray data

Raw microarray data was processed and analyzed with the Bioconductor aroma package (Bengtsson et al., 2008) and normalized using the RMA method from the Bioconductor Affy package. The quantile normalization step of the RMA normalization was performed at the probeset level. Using the normalized data, genes with significant evidence for differential expression were identified with the limma package (Smyth, 2004) in Bioconductor. The limma methodology calculates a *p*-value for each gene using a modified *t*-test in conjunction with an empirical Bayesian method to moderate the standard errors of the estimated log-fold changes. This method of detecting differentially expressed genes draws strength across genes for more robust and accurate detection of differentially expressed genes. Such an adjustment has repeatedly been shown to avoid an excess of false positives when identifying differentially expressed genes (Allison et al., 2006). Using the *p*-values from limma, we used the Bioconductor package p.adjust (Benjamini and Hochberg, 1995) to estimate the false discovery rate associated with our list of differentially expressed genes. This methodology allows us to address the multiple testing problem without resorting to an excessively conservative approach that controls the familywise error, such as a Bonferroni correction. Venn diagrams were generated with the Bioconductor limma package.

Ingenuity Pathway Analysis (IPA; Build 131235; Version 11904312; Database Status 02.20.2012)^2^ was used to analyze differentially expressed genes (>1.5-fold up or down-regulated, *p* < 0.05) using the Core Analysis feature. IPA is a commercial tool that is based on a proprietary database (see text footnote 2) to facilitate the identification of biological themes, in microarray gene expression data. IPA uses a Right-tailed Fisher’s exact test to calculate a *p*-value determining the probability that each biological function, canonical pathway, or transcriptional network assigned to the data set is due to chance alone.

### Validation of data obtained with microarrays using fluorogenic 5′-nuclease-based assay and quantitative RT-PCR

Quantitative TaqMan based RT-PCR (qPCR) analysis has a greater dynamic range for changes in gene expression levels compared to microarray-based analysis. Therefore, we used quantitative-PCR (qPCR) to validate expression changes of 15 genes of interest that had been identified by microarray analysis with significant changes by either TBI or nicotinamide treatment. Briefly, reverse transcription was performed according to the manufacturer’s established protocol using total RNA and the SuperScript^®^ III First-Strand Synthesis System (Invitrogen, Carlsbad, CA, USA). For gene expression measurements, 2 μL of cDNA were included in a PCR reaction (12 μL final volume) that also consisted of the ABI inventoried TaqMan^®^ Gene Expression Assays mix and the TaqMan Gene Expression Master Mix according to the manufacturer’s protocol (Applied Biosystems, Inc., Foster City, CA, USA). Amplification and detection of PCR amplicons were performed with the ABI PRISM 7900 system (Applied Biosystems, Inc., Foster City, CA, USA) with the following PCR reaction profile: 1 cycle of 95°C for 10 min, 40 cycles of 95°C for 30 s, and 60°C for 1 min. β-Actin amplification plots derived from serial dilutions of an established reference sample were used to create a linear regression formula in order to calculate expression levels. β-Actin gene expression levels were utilized as an internal control to normalize the data.

## Results

### Nicotinamide concentrations

In the healthy animals, 75 mg/kg s.c. bolus plus an infusion rate of 12.0 mg/h kg of nicotinamide resulted in peak concentrations of 68 ± 8 μg/mL at 1 h with steady state concentrations of 41 ± 7, 36 ± 8, and 33 ± 9 μg/mL at 24, 48, and 72 h post-infusion, respectively. In the CCI injured animals, nicotinamide concentrations were 45 ± 12 and 55 ± 4 μg/mL at 24 and 72 h, respectively at time of sacrifice. The nicotinamide concentrations was 50 ± 13 μg/mL obtained by tail snip at 72 h at the time of removal of the osmotic pump in the animals sacrificed at 7 days. Nicotinamide concentrations were below the detectable limit at the time of sacrifice at 7 days post-CCI.

### Gene expression study

**Figure 2:**
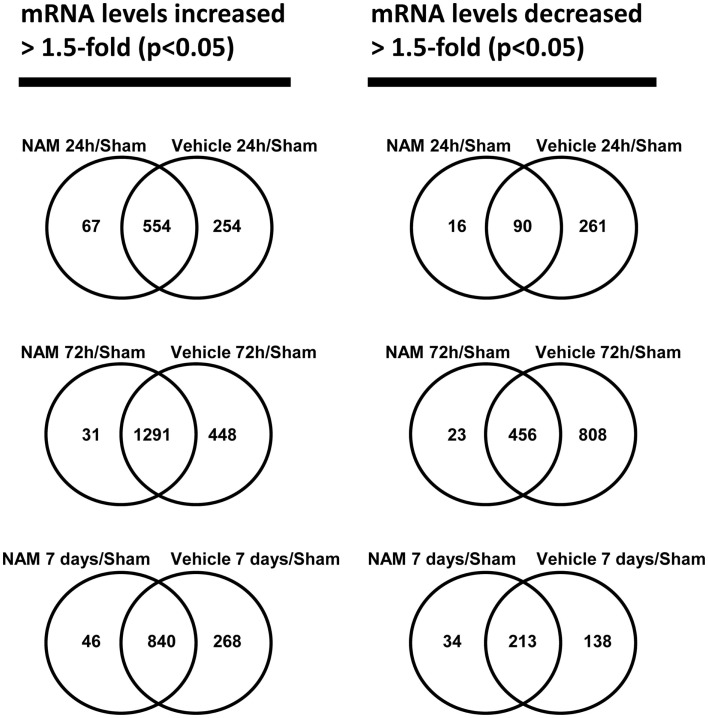
**The Venn diagrams show the number of genes whose expression was up or down-regulated more than 1.5-fold (*p* < 0.05) in the NAM/Sham and Vehicle/Sham contrasts at the 24 h, 72 h, and 7-day time points**. Venn diagrams were generated with the Bioconductor limma package

The microarray data passed all the standard and advanced quality control metrics. The number of differentially expressed genes (>1.5-fold change, *p* < 0.05) at 24 h, 72 h, and 7 days is presented in Table 1. The vehicle to sham (no injury) comparison reflects the effect of the TBI without treatment relative to sham controls. At 24 h, 72 h, and 7 days, respectively, 70, 58, and 76%, of the differentially expressed genes were up-regulated in the vehicle treated animals compared to the sham animals (Table 1). The nicotinamide to sham comparison reflects the effect of TBI with treatment compared to sham controls. The Venn diagrams show the number of genes differentially expressed more than 1.5-fold (*p* < 0.05) in the nicotinamide compared to sham and vehicle compared to sham contrasts at the 24-h, 72 h, and 7 day time points (Figure 2). At 24 h, vehicle treatment resulted in increased expression of 808 genes (>1.5-fold, *p* < 0.05) compared to non-injured sham animals, whereas only 621 genes in the nicotinamide treated animals showed elevated expression. Similarly, at 24 h post-TBI, mRNA levels of 351 genes were decreased more than 1.5-fold (*p* < 0.05) compared to non-injured sham animals, whereas only 106 genes exhibited decreased expression in the nicotinamide treated animals. At 72 h, vehicle treatment resulted in increased expression of 1739 genes (>1.5-fold, *p* < 0.05) compared to non-injured sham animals, whereas only 1332 genes in the nicotinamide treated animals showed elevated expression. In the same way, 72 h post-TBI, mRNA levels of 1264 genes were decreased more than 1.5-fold (*p* < 0.05) compared to non-injured sham animals, whereas only 479 genes exhibited decreased expression in the nicotinamide treated animals. By 7 day post-TBI, vehicle treatment resulted in increased expression of 1108 genes (>1.5-fold, *p* < 0.05) compared to non-injured sham animals, whereas only 886 genes in the nicotinamide treated animals showed elevated expression. Likewise, mRNA levels of 351 genes were decreased more than 1.5-fold (*p* < 0.05) compared to non-injured sham animals, whereas only 247 genes exhibited decreased expression in the nicotinamide treated animals. Overall, the Venn diagrams demonstrate the effect of nicotinamide on counteracting the effect of TBI which results in significantly decreased number of genes differentially expressed by TBI.

**Table 1:** **Brain gene expression data**.

	24 h			72 h			7 days		
	Down	Up	Total	Down	Up	Total	Down	Up	Total
Vehicle/sham	351	808	1159	1264	1739	3002	351	1108	1459
NAM/sham	106	621	727	479	1322	1801	247	886	1133
NAM/vehicle	124	26	150	12	107	119	4	1	5

*The number of differentially expressed genes (>1.5-fold up or down, *p* < 0.05)*.

The nicotinamide (CCI animals that received nicotinamide) to vehicle (CCI animals that received vehicle) comparison evaluates the effect of nicotinamide treatment by separating out the effect of the TBI on gene expression. At 24 h post-TBI, the majority (82%) of the 150 differentially expressed genes in the nicotinamide treated animals compared to vehicle were down-regulated (Table 1). At 72 h post-TBI, there were 119 differentially expressed genes in the nicotinamide treated animals compared to vehicle with the majority (90%) being up-regulated. At 7 days post-TBI (4 days post-nicotinamide treatment), there were only five differentially expressed genes in the nicotinamide compared to vehicle treated animals.

**Figure 3:**
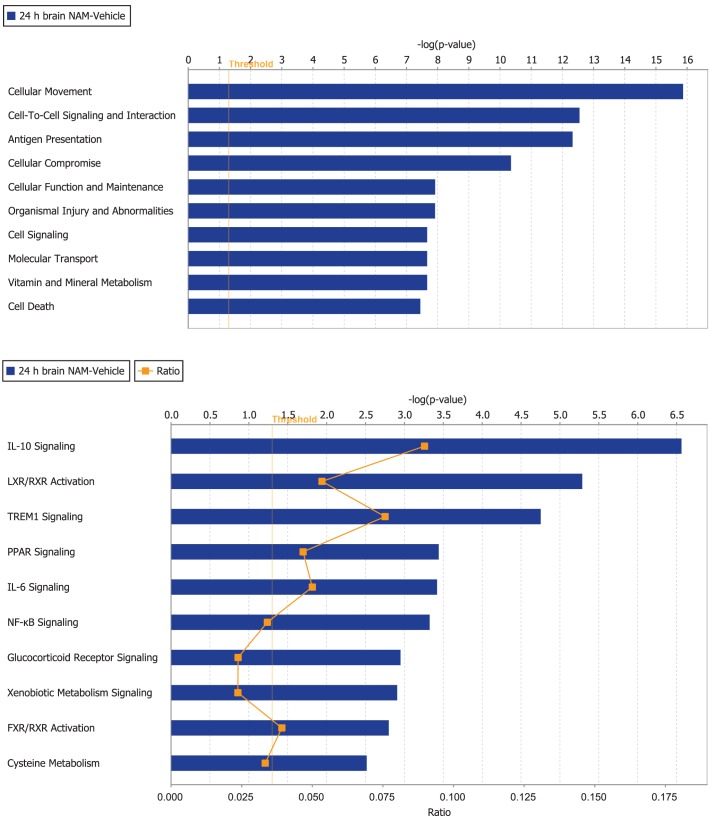
**The top 10 functional categories and canonical pathways involving significantly regulated genes in the nicotinamide treated compared to vehicle treated CCI injured animals for the 24-h time point**. The length of the bar is the negative log of the *p*-value and is significant if it extends to the right of the threshold line −log(*p* < 0.05). The ratio number, is defined as the number of significant molecules in the given pathways divided by the total number of molecules that are included in the pathway.

**Figure 4:**
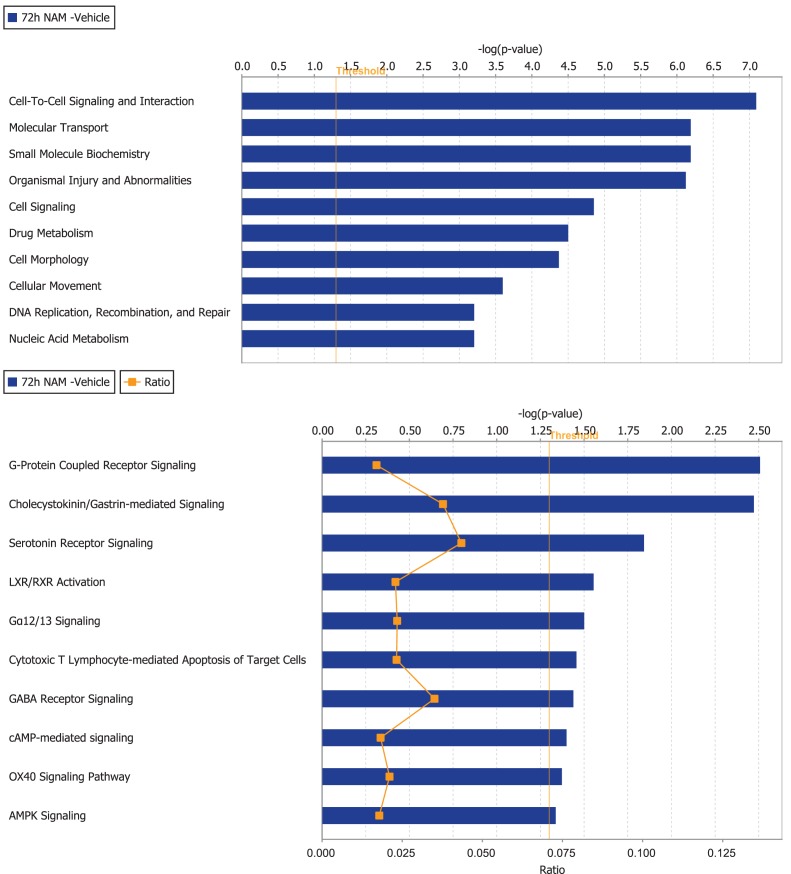
**The top 10 functional categories and canonical pathways involving significantly regulated genes in the nicotinamide treated compared to vehicle treated CCI injured animals for the 72-h time point**. The length of the bar is the negative log of the *p-*value and is significant if it extends to the right of the threshold line [−log(*p* < 0.05)]. The ratio number, is defined as the number of significant molecules in the given pathways divided by the total number of molecules that are included in the pathway.

**Figure 5:**
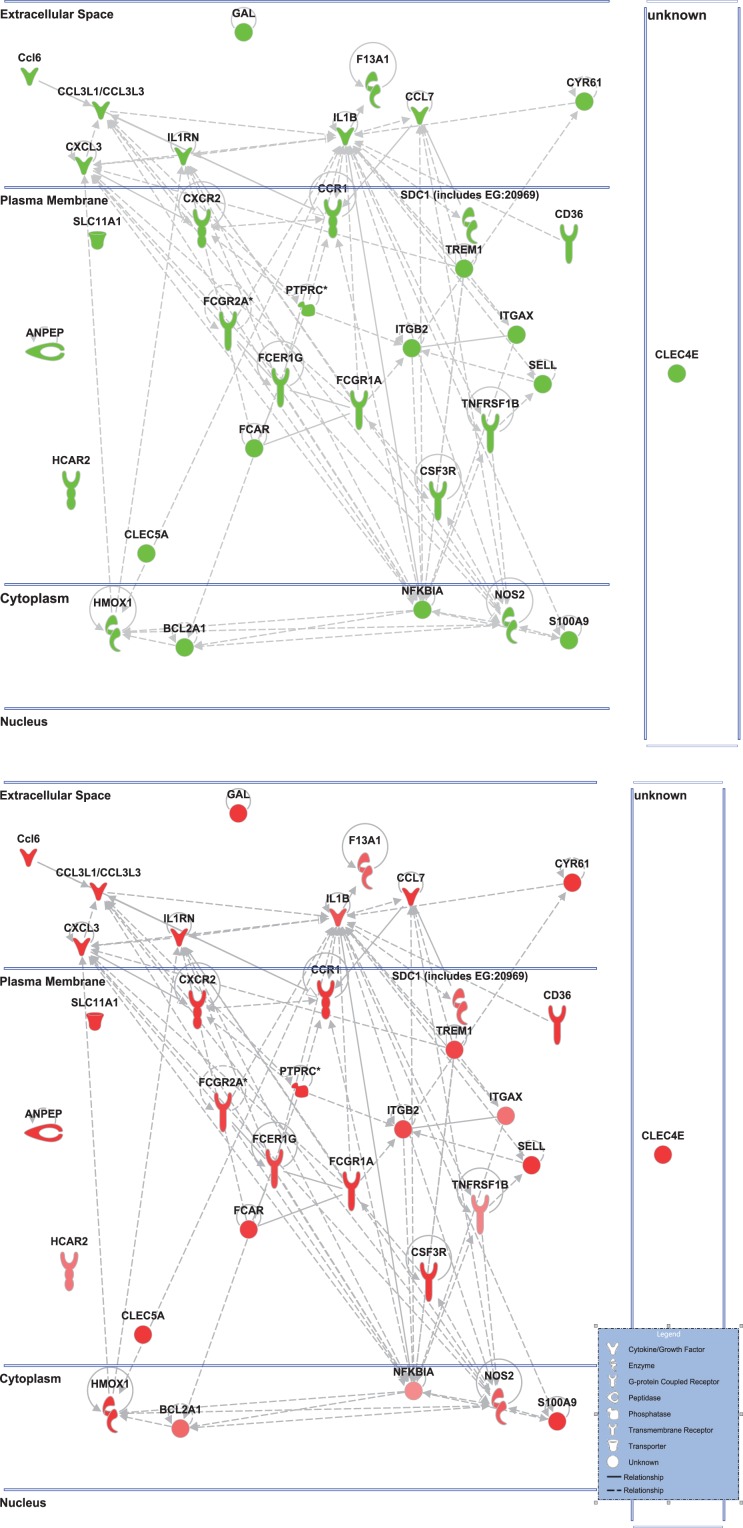
**Cell-to-cell signaling network generated by ingenuity pathway analysis of differentially expressed genes: Molecules are represented as nodes, and the biological relationship between two nodes is represented as an edge (line)**. All edges are supported by at least one reference from the literature, from a textbook, or from canonical information stored in the Ingenuity Knowledge Base. The intensity of the node color indicates the degree of up- (red) or down- (green) regulation. Nodes are displayed using various shapes that represent the functional class of the gene product. Edges are displayed with various labels that describe the nature of the relationship between the nodes. Top: the nicotinamide treated injured animals compared to vehicle treated injured animal. Bottom: the vehicle treated injured animals compared to sham controls highlighting only the genes affected by nicotinamide treatment shown in the top figure.

Ingenuity Pathway Analysis was used to facilitate the identification of biological themes in the microarray data. IPA shifts the emphasis from the evaluation of single genes to an evaluation of molecular pathways, networks, and biological functions. Functional categories were identified by molecular and cellular functions. Canonical pathways include signaling and metabolic pathways. IPA was used to generate Figures 3–5; Figures 3 and 4 show bar-graphs for the top 10 functional categories and canonical pathways for the NAM/Vehicle comparison ranked according to statistical significance (*p* < 0.05). Figure 5 depicts the second most significant functional category, i.e., the Cell-to-Cell-Signaling and Interaction network (*p* < 0.05) in detail; IPA identified 24 and 27 significant (*p* < 0.05) functional categories, and 120 and 81 significant (*p* < 0.05) canonical pathways at 24 and 72 h post-TBI for the NAM/vehicle contrasts, respectively. The differentially expressed genes associated with each of the top 10 functional categories and canonical pathways are listed in Tables 2 and 3, whereas their degree of differential expression for the NAM/Vehicle and Vehicle/Sham contrasts at 24 and 72 h are provided in Tables 4 and 5 respectively.

**Table 2:** **Regulated genes in the functional categories and canonical pathways at 24 h post-TBI**.

**Functional categories**	
Cellular movement	Fcar, Trem1, Sell, Anpep, Adam8, Ptprc, Hmox1, Cxcl3, Nfkbia, Cxcr2, Tnfrsf1b, Nos2, Hcar2, Ccl6, Ccr1, Csf3r, Sdc1, Fcgr2a, Cd36, Alox5ap, F13a1, Itgb2, Clec4e, S1009, Ccl7, Il1rn, Fcer1g, Il1b, Ccl3l1/Ccl3l3, Cyr61, Itgax, Arg1
Cell-to-cell signaling and interaction	Fcar, Sell, Trem1, Anpep, Fcgr1a, Ptprc, Cxcl3, Hmox1, Nfkbia, Gal, Cxcr2, Tnfrsf1b, Nos2, Ccl6, Hcar2, Csf3r, Ccr1, Sdc1, Fcgr2A, Cd36, F13a1, Clec5a, Itgb2, Ccl7, Clec4e, S100A9, Il1rn, Fcer1G, Ccl3l1/Ccl3l3, Il1b, Bcl2a1, Cyr61, Slc11a1, Itgax
Antigen presentation	Fcar, Trem1, Sell, Fcgr1a, Anpep, Cxcl3, Hmox1, Cxcr2, Tnfrsf1b, Nos2, Hcar2, Csf3r, Ccr1, Sdc1, Fcgr2a, Cd36, Clec5a, Itgb2, Clec4e, S100a9, Ccl7, Il1rn, Fcer1g, Il1b, Ccl3l1/Ccl3l3, Slc11a1, Itgax, Arg1
Cellular compromise	Ccr1, Fcar, Gsta2, Sell, Trem1, Sdc1, Fcgr2a, Anpep, Ptprc, Itgb2, Cxcl3, Hmox1, Sl00a9, Il1rn, Pilra, Fcer1g, Ccl13l/Ccl3l3, Il1b, Tnfrsf1b, Nos2, Slc11a1, Arg1
Cellular function and maintenance	Csf3r, Fcar, Ccr1, Sell, Trem1, Fcgr2a, Cd36, Anpep, Fcgr1A, Ptprc, Itgb2, Cxcl3, Ccl7, Fcer1g, Ccl3l1/Ccl3l3, Il1b, Slc11a1
Organismal injury and abnormalities	Sell, Trem1, Anpep, Fcgr1a, Il1r2, Hmox1, Nfkbia, Hbb, Gal, Cxcr2, Nos2, Tnfrsf1b, Ccl6, Csf3r, Slpi, Fcgr2a, Cd36, F13a1, Itgb2, Sl00a9, Il1rn, Fcer1g, Il1b, Ccl13l/Ccl3l3, Arg1
Cell signaling	Sell, Trem1, Igsf6, Fcgr1a, Ptprc, Il1r2, Cxcl3, Hmox1, Gal, Cxcr2, Tnfrsf1b, Nos2, Ccr1, Fcgr2a, Cd36, Hhip, Itgb2, Ccl7, S100a9, Il1rn, Fcer1g, Pilr2, Il1b, Ccl3l1/Ccl3l3, Arg1
Molecular transport	Trem1, Sell, Fcgr1a, Ptprc, Il1r2, Cxcl3, Hmox1, Nfkbia, Slc6a20, Gal, Cxcr2, Plin2, Nos2, Tnfrsf1b, Hcar2, Ccr1, Fcgr2a, Cd36, Alox5ap, Sgms2, Itgb2, Ccl7, S100a9, Il1rn, Pilra, Fcer1g, il1b, Ccl3l1/Ccl3l3, Slc11a1, Arg1
Vitamin and mineral metabolism	Ccr1, Trem1, Sell, Fcgr2a, Fcgr1a, Ptprc, Itgtb2, Cxcl3, Ccl7, S100a9, Gal, Crcx2, Pilra, Fcer1g, Ccl3l1/Ccl3l3, Il1b, Nos2
Cell death	Fcar, Trem1, Glipr1, Anpep, Fcgr1a, Adam8, Ptprc, Hmox1, Cxcl3, Hbb, Nfkbia, Gal, Cxcr2, Nos2, Tnfrsf1b, Ccl6, Arl11, Hcar2, Csf3r, Cd53, Ccr1, Gsta2, Sdc1, Fcgr2a, Cd36, F13a1, Sgms2, Clec5a, Itgb2, S100a9, Ccl7, Il1rn, Fcer1g, Ccl3l1/Ccl3l3, Brca2, Il1b, Bcl2a1, Cyr61, Arg1
**Canonical pathway**	
Il-10 signaling	Il1r2, Ccr1, Hmox1, Nfkbia, Fcgr2a, Il1rn, Il1b
LXR/RXR activation	Il1r2, Ccl7, Il1rn, Cd36, Il1b, Tnfrsf1b, Nos2
TREM1 signaling	Trem1, Ccl7, Tlr13, Il1b, Itgax
Communication between innate and adaptive immune cells	Il1rn, Fcer1g, Ccl13l/Ccl3l3, Tir13, Il1b
PPAR signaling	Il1r2, Nfkbia, Il1rn, Il1b, Tnfrsf1b
Il-6 signaling	Il1r2, Nfkbia, Il1rn, Il1b, Tnfrsf1b
NF-κB signaling	Il1r2, Nfkbia, Il1rn, Fcer1g, Il1b, Tnfrsf1b
Glucocorticoid receptor signaling	Il1r2, Cxcl3, Nfkbia, Il1rn, Il1b, Nos2, Fcgr1a
Xenobiotic metabolism signaling	Hmox1, Gsta2, Il1b, Sult1d1, Nos2, Sult2a1
FXR/RXR activation	Sdc1, Il1rn, Il1b, Sult2a1

**Table 3:** **Regulated genes in the functional categories and canonical pathways at 72 h post-TBI**.

**Functional pathways**	
Cell-to-cell signaling and interaction	Cacna1g, Kcnh1, Scn2a, Sv2b, Egr3, Cacnb4, Rgs4, Cck, Stx1a, Hrh3, Efna5, Sst, Htr1f, Ptgs2, Chrna5, Adra1b, Gabra3, Mmp9, Agt, Htr2a
Molecular transport	Cacna1g, Kcnh1, Clic5, Scn2a, Slc4a11, Cacnb4, Rgs4, Kcnip3, Cck, Stx1a, Hrh3, Kcnh7, Sst, Lyve1, Ptgs2, Chrna5, Gabre, Adra1b, Kcng3, Atp2b4, Agt, Htr2a
small molecular biochemistry	Kcnh1, Rxfp2, Cacnb4, Sstr1, Rgs4, Kcnip3, Cck, Stx1a, Pde1a, Hrh3, Acaa2, Ephb6, Gpx3, Tph1, Sst, Ptgs2, Lyve1, Chrna5, Atp2b4, Adra1b, Htr2a, Agt
Organismal injury and abnormalities	Cacna1l, Cacna1g, Kcnip3, Htr1f, Ptgs2, Chrna5, Gabre, Adra1b, Mmp9, Gabra3, Agt, Htr2a
Cell signaling	Cacna1g, Rxfp2, Cacnb4, Rgs4, Sstr1, Kcnip3, Cck, Ped1a, Hrh3, Gnat2, Sst, Htr1f, Ptgs2, Adra1b, Atp2b4, Htr2a, Agt
Drug metabolism	Sst, Rgs4, Lyve1, Cck, Ptgs2, Chrna5, Stx1a, Hrh3, Adra1b, Agt, Htr2a
Cell morphology	Efna5, Sst, Rgs4, Ptgs2, Adra1b, Mmp9, Agt
Cellular movement	Satb2, Sst, Rgs4, Tbr1, Ptgs2, Mmp9, Agt
DNA replication, recombination, and repair	Sst, Ptgs2, Ped1a, Agt
Nucleic acid metabolism	Ephb6, Rxfp2, Cacnb4, Sstr1, Sst, Cck, Ptgs2, Ped1a, Hrh3, Acaa2, Atp2b4, Agt
**Canonical pathway**	
G-protein couple receptor signaling	Rxfp2, Rgs4, Sstr1, Htr1f, Tacr3, Pde1a, Hrh3, Adra1b, Htr2a
Cholecystokinin/gastrin-mediated signaling	Sst, Mef2c, Cck, Ptgs2
Serotonin receptor signaling	Tph1, Htr2a
LXR/RXR activation	Ptgs2, Mmp9, Agt
Gα12/13 signaling	Cdh7, Cdh12, Mef2c
Cytotoxic T lymphocyte-mediated apoptosis of target cells	Hla-Dq1a, RT1-M6-1/Rt1-M6-2
GABA receptor signaling	Gabre, Gabra3
c-AMP-mediated signaling	Rgs4, Htr1f, Pde1a, Hrh3
OX40 signaling pathway	Hla-Dq1a, RT1-M6-1/Rt1-MG-2
AMPK signaling	Pfkp, Chrna5, Adra1b

**Table 4:** **The effect of NAM treatment (NAM/vehicle) and TBI (vehicle/sham) on differentially expressed genes associated with significant functional categories and canonical pathways identified by IPA at 24 h post-TBI**.

Affymetrix ID	Gene symbol	Genes	24 h post-TBI		72 h post-TBI	
			NAM/vehicle	Vehicle/sham	NAM/vehicle	Vehicle/sham
10726550	Adam8	ADAM metallopeptidase domain 8	0.59	2.11	n.s.	2.76
10759999	Alox5ap	Arachidonate 5-lipoxygenase activating protein	0.64	3.28	n.s.	3.85
10722992	Anpep	Alanyl (membrane) aminopeptidase	0.43	6.51	n.s.	10.18
10702214	Arg1	Arginase	0.66	1.78	n.s.	n.s.
10780919	Arl11	ADP-ribosylation factor-like 11	0.58	3.74	n.s.	6.69
10797660	Aspn	Asporin	1.52	n.s.	n.s.	n.s.
10912112	Bcl2a1	BCL2-related protein A1	0.63	2.65	n.s.	5.48
10759834	Brca2	Breast cancer 2	0.61	1.66	n.s.	n.s.
10745677	Ccl13l/Ccl3l3	Chemokine (C–C motif) ligand 3-like 1	0.49	6.41	n.s.	3.39
10745670	Ccl6	Chemokine (C–C motif) ligand 6	0.42	6.19	n.s.	3.73
10736702	Ccl7	Chemokine (C–C motif) ligand 7	0.67	4.39	n.s.	3.35
10921163	Ccr1	Chemokine (C–C motif) receptor 1	0.53	4.11	n.s.	2.42
10853240	Cd36	CD36 molecule (thrombospondin receptor)	0.57	5.26	n.s.	7.57
10825809	Cd53	Cd53 molecule	0.61	3.99	n.s.	8.89
10865381	Clec4e	C-type lectin domain family 4, member e	0.39	10.51	n.s.	2.66
10862131	Clec5a	C-type lectin domain family 5, member A	0.66	4.11	n.s.	3.15
10871957	Csf3r	Colony stimulating factor 3 receptor	0.55	4.17	n.s.	3.72
10775896	Cxcl3	Chemokine (C-X-C motif) ligand 3	0.41	5.11	n.s.	n.s.
10924245	Cxcr2	Chemokine (C-X-C motif) receptor 2	0.51	5.77	n.s.	1.65
10787197	Cyp4f18	Cytochrome P450, family 4, subfamily 5, polypeptide 18	1.54	0.54	n.s.	n.s.
10827231	Cyr61	Cysteine-rich, angiogenic inducer, 61	0.66	3.95	n.s.	1.50
10794734	F13a1	Coagulation factor XIII, A1 polypepetide	0.54	2.81	n.s.	3.41
10718934	Fcar	IgA Fc receptor	0.49	3.51	n.s.	n.s.
10769825	Fcer1g	Fc fragment of IgE, high affinity I, receptor for; gamma polypeptide	0.62	3.39	n.s.	6.00
10825153	Fcgr1a	Fc fragment of IgG, high affinity Ia, receptor	0.59	3.78	n.s.	4.23
10769788	Fcgr2a	Fc fragment of IgG, low affinity IIa, receptor	0.53	3.44	n.s.	4.44
10727321	Gal	Galaninprepropeptide	0.63	4.25	n.s.	2.59
10902313	Glipr1	GLI pathogenesis-related 1	0.65	4.33	n.s.	5.43
10926967	Gsta2	Glutathione *S*-transferase alpha 2	1.63	0.42	n.s.	n.s.
10724311	Hbb	Hemoglobin, beta	0.62	2.91	n.s.	1.71
10758351	Hcar2	Hydroxycarboxylic acid receptor 2	0.56	2.39	n.s.	n.s.
10806946	Hhip	Hedgehog-interaction protein	1.56	0.46	n.s.	n.s.
10806122	Hmox1*	Heme oxygenase (decycling) 1	0.56	8.47	n.s.	11.60
10761128	Hspb1*	Heat shock protein	0.54	3.19	n.s.	1.51
10725397	Igsf6	Immunoglobulin superfamily, member 6	0.60	4.52	n.s.	3.87
10849841	Il1b*	Interleukin 1 beta	0.62	3.10	n.s.	n.s.
10922816	Il1r2	Interleukin 1 receptor, type II	0.49	3.81	n.s.	n.s.
10834109	Il1rn*	Interleukin 1 receptor antagonist	0.58	5.58	n.s.	4.88
10711299	ltgax	Integrin, alpha X	0.64	2.46	n.s.	2.19
10832306	Itgb2	Integrin, beta 2	0.63	3.27	n.s.	5.99
10890024	Nfkbia	Nuclear factor of kappa light polypeptide gene enhancer in B-cells inhibitor, alpha	0.63	2.08	n.s.	1.68
10736312	Niacr1	Niacin receptor 1	0.56	2.39	n.s.	n.s.
10736312	Nos2	Nitric oxide synthase 2, inducible	0.56	2.91	n.s.	n.s.
10761025	Pilra	Paired immunoglobulin-like type 2 receptor alpha	0.64	3.07	n.s.	2.90
10877907	Plin2	Perilipin 2	0.50	6.08	n.s.	6.17
10768138	Ptprc	Protein tyrosine phosphatase, receptor type, C	0.66	4.62	n.s.	9.39
10824695	S100a9*	S100 calcium binding protein A9	0.52	3.73	n.s.	1.55
10883530	Sdc1	Syndecan 1	0.66	2.94	n.s.	2.25
10765186	Sell	Selectin L	0.51	4.70	n.s.	2.11
10826846	Sgms2	Sphingomyelin synthase 2	0.61	2.27	n.s.	2.46
10921120	Slc6a20	Solute carrier family 6 (proline IMINO transporter), member 20	1.70	1.99	n.s	2.72
10924286	Slc11a1	Solute carrier family 11 (proton-coupled divalent metal ion transporters), member 1	0.60	3.76	n.s.	6.02
10851581	Slpi	Secretory leukocyte peptidase inhibitor	0.42	4.29	n.s.	3.41
10771919	Sult1d1	Sulfotransferase family 1D, member 1	1.53	0.48	n.s.	0.65
10719187	Sult2a1	Sulfotransferase family 2A, dehydroepiandrosterone (DHEA)-preferring, member 1	0.66	n.s.	n.s.	n.s.
10934608	Tlr13	Toll-like receptor 13	0.61	3.02	n.s.	5.84
10881424	Tnfrsf1b*	Tumor necrosis factor receptor superfamily, member 1b	0.64	2.22	n.s.	2.84
10926277	Trem1	Triggering receptor expressed on myeloid cells 1	0.58	3.35	n.s.	5.43

*Results are given as fold change with significance defined as 1.5-fold change, *p* < 0.05. The degree of differential expression of these genes is also shown for the NAM/Vehicle and Vehicle/Sham contrasts for the 72-h post-TBI time point*.

**Figure 6:**
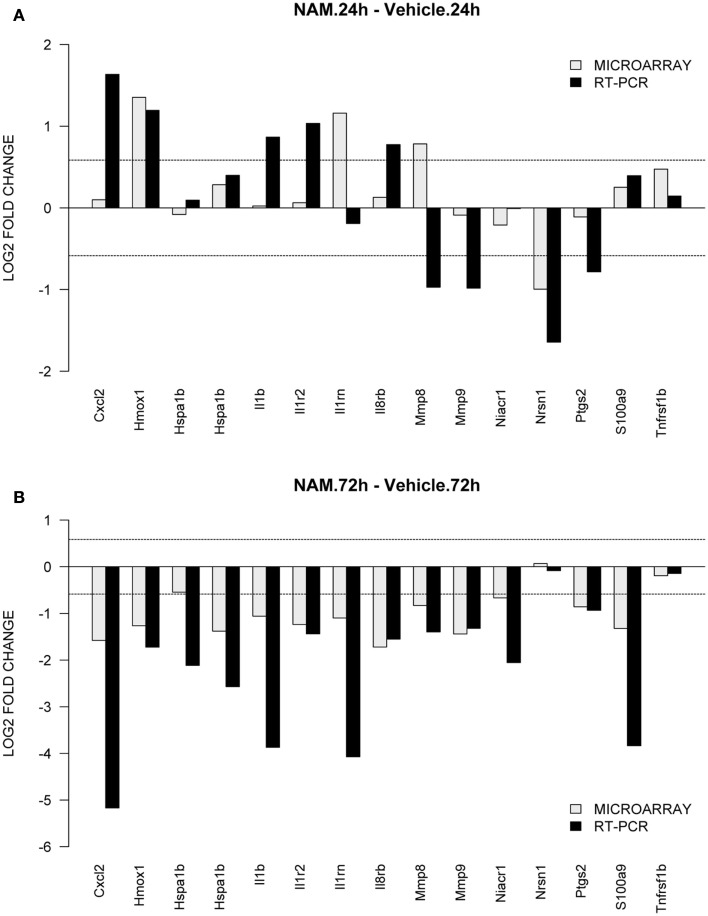
**TaqMan based RT-PCR validation of the microarray data for the selected genes: Cxc12, chemokine (C-X-C motif) ligand 2; Hmox1, heme oxygenase 1; Hspa1b, heat shock protein 1b; Il1b, interleukin 1beta; Il1rn, interleukin 1 receptor antagonist; Il8rb, interleukin 8 receptor beta; Mmp8, matrix metallopeptidase 8; Mmp9, matrix metallopeptidase 9; Niacr1, niacin receptor 1; Nrsn1, neurensin 1; Ptgs2, prostaglandin-endoperoxide synthase 2; S100a9, S100 calcium binding protein A9; Tnfrsf1b, tumor necrosis factor receptor superfamily, member 1b**. The RT-PCR data was normalized to the housekeeping gene β-actin. In order to compare the microarray data to the RT-PCR data, the microarray data for each gene was divided by the beta-actin data as it was measured by the microarray analysis. The gray bars show the microarray data and the black bars display the RT-PCR data for the contrast. **(A,B)** Show the data from the 24 and 72-h time point for the contrasts nicotinamide treated injured animals compared to the vehicle treated injured animals.

*RT-PCR validated (see Figure 6).

*n.s., not significant*.

**Table 5:** **The effect of NAM treatment (NAM/vehicle) and TBI (vehicle/sham) on differentially expressed genes associated with significant functional categories and canonical pathways identified by IPA at 24 h post-TBI**.

Affymetrix ID	Gene symbol	Genes	24 h post-TBI		72 h post-TBI	
			NAM/vehicle	Vehicle/sham	NAM/vehicle	Vehicle/sham
10802691	Acaa2	Acetyl-Coenzyme A acyltransferase 2	n.s.	n.s.	0.66	2.29
10742213	Adra1b	Adrenergic, alpha-1B-, receptor	n.s.	n.s.	1.53	0.49
10811900	Agt	Angiotensinogen (serpin peptidase inhibitor, clade A, member 8)	n.s.	0.55	0.66	n.s.
10767723	Atp2b4	ATPase, Ca++ transporting, plasma membrane 4	n.s.	n.s.	1.61	0.53
10746327	Cacna1g	Calcium channel, voltage-dependent, T type, alpha 1G subunit	n.s.	n.s.	1.56	0.57
10845306	Cacnb4	Calcium channel, voltage-dependent, beta 4 subunit	n.s	n.s.	1.55	0.43
10921030	Cck	Cholecystokinin	n.s.	n.s.	2.27	0.38
10763401	Cdh7	Cadherin 7, type 2	n.s.	n.s.	1.55	0.38
10813858	Cdh12	Cadherin 12	n.s.	0.64	1.89	0.26
10910133	Chrna5	Cholinergic receptor, nicotinic, alpha 5	n.s.	n.s.	3.22	n.s.
10926642	Clic5	Chloride intracellular channel 5	n.s.	n.s.	1.52	n.s.
10736697	Ccl2*	Chemokine (C-C motif) ligand 2	n.s.	44.15	n.s.	36.50
10930204	Efna5	Ephrin A5	n.s.	n.s.	2.28	0.27
10781337	Egr3	Early growth response 3	n.s.	n.s.	1.61	0.51
10854961	Ephb6	Eph receptor B6	3.09	n.s.	1.63	0.50
10909987	Exph5	Exophilin 5	n.s.	n.s.	1.60	0.31
10940090	Gabra3	Gamma-aminobutyric acid (GABA) A receptor, alpha 3	n.s.	n.s.	1.56	0.46
10935811	Gabre	Gamma-aminobutyric acid (GABA) A receptor, epsilon	n.s.	1.52	1.50	n.s.
10818306	Gnat2	Guanine nucleotide binding protein, alpha transducing 2	n.s.	n.s.	0.61	1.81
10733680	Gpx3	Glutathione peroxidase	n.s.	n.s.	0.67	2.21
10871939	Grik3	Glutamate receptor, ionotropic, kainite 3	n.s.	n.s.	1.67	0.47
10828351	HLA-DQA1	Major histocompatibility complex, class II, DQ alpha 1	n.s	1.65	0.62	3.58
10852258	Hrh3	Histamine receptor H3	n.s.	n.s.	1.58	0.54
10752663	Htr1f	5-Hydroxytryptamine (serotonin) receptor 1F	n.s.	0.66	1.59	0.39
10781467	Htr2a	5-Hydroxytryptamine (serotonin) receptor 2A	n.s.	n.s.	2.37	0.31
10889326	Kcnf1	Potassium voltage-gated channel, subfamily F, member 1	n.s.	n.s.	1.68	0.33
10888131	Kcng3	Potassium voltage-gated channel, subfamily G, member 3	n.s.	n.s.	1.52	0.57
10845725	Kcnh7	Potassium voltage-gated channel, subfamily H (eag-related), member 7	n.s.	0.65	1.51	0.34
10849655	Kcnip3	Kv channel interacting protein 3, calsenilin	n.s.	n.s.	2.94	n.s.
10724895	Lyve1	Lymphatic vessel endothelial hyaluronan receptor 1	n.s.	n.s.	0.58	3.37
10820223	Mef2c	Myocyte enhancer factor 2C	n.s.	n.s.	1.78	0.38
10907913	Mmp8*	Matrix metallopeptidase 8	n.s.	3.24	n.s.	4.15
10842239	Mmp9*	Matrix metallopeptidase 9	n.s.	3.36	1.60	n.s.
10795077	Nrsn1*	Neurensin 1	n.s.	n.s.	2.99	0.39
10782986	Otx2	Orthodenticle homeobox 2	n.s.	n.s.	0.41	3.37
10846694	Ped1a	Phosphodiesterase 1A, calmodulin-dependent	n.s.	n.s.	3.68	0.44
10795989	Pfkp	Phosphofructokinase, platelet	n.s.	n.s.	1.70	0.59
10764551	Ptgs2*	Prostaglandin-endoperoxide synthase 2	n.s.	1.85	1.72	n.s.
10846694	Pde1a	Phosphodiesterase 1A, calmodulin-dependent	n.s.	n.s.	1.88	0.44
10744641	Pitpnm3	PITPNM family member 3	n.s.	n.s.	1.57	0.48
10769672	Rgs4	Regulator of G-protein signaling 4	n.s.	n.s.	1.82	0.30
10827686	RT1-M6-1	RT1 class I, locus M6, gene 1	n.s.	n.s.	2.16	0.50
10827691	RT1-M6-2	RT1 class I, locus M6, gene 2	n.s.	n.s.	1.89	0.62
10759923	Rxfp2	Relaxin/insulin-like family peptide receptor 2	n.s.	0.66	1.51	0.65
10836458	Scn2a	Sodium channel, voltage-gated, type II, alpha subunit	n.s.	n.s	1.50	0.46
10921274	Satb1	SATB homeobox 1	n.s.	n.s.	1.52	0.42
10928191	Satb2	SATB homeobox 2	n.s.	n.s.	2.50	0.24
10849943	Slc4a11	Solute carrier family 4, sodium bicarbonate transporter-like, member 11	n.s.	n.s.	1.56	0.65
10751945	Sst	Somatostatin	n.s.	n.s.	1.57	0.49
10884732	Sstr1	Somatostatin receptor 1	n.s.	n.s.	1.64	0.43
10930711	St6gal2	ST6 beta-galactosamide α-2,6-siayltransferase 2	n.s.	0.66	1.81	0.45
10802795	St8sia5	ST8 alpha-*N*-acetyl-neuraminide α-2,8-sialyltransferase 5	n.s.	n.s.	1.61	0.44
10763171	Stx1a	Syntaxin 1A (brain)	n.s.	n.s.	2.16	0.32
10722830	Sv2b	Synaptic vesicle glycoprotein 2b	n.s.	n.s.	1.81	0.43
10819139	Tacr3	Tachykinin receptor 3	n.s.	0.50	1.59	0.29
10845628	Tbr1	T-box, brain, 1	n.s.	n.s.	1.90	0.32
10722097	Tph1	Tryptophan hydroxylase 1	n.s.	n.s.	0.47	2.23

*Results are given as fold change with significance defined as 1.5-fold change, *p* < 0.05. The degree of differential expression of these genes is also shown for the NAM/Vehicle and Vehicle/Sham contrasts for the 24-h post-TBI time point*.

*RT-PCR validated (see Figure 6).

*n.s., not significant*.

At 24 h post-TBI, 58 unique genes were over-represented in the IPA identified significant functional categories and canonical pathways in the NAM/vehicle contrast with many genes included in multiple categories (Table 4). The majority of the regulated genes was involved in cell movement, cell signaling, cell death, and the inflammatory/immune system and included several cytokines, cytokine receptors, and chemokines as well as other molecules involved in the inflammatory/immune response. The expression of the majority of the regulated genes down-regulated by nicotinamide, were up-regulated by TBI as shown in Table 4. At 72 h, there was still significantly increased expression in the vehicle treated injured compared to non-injured sham animals in the majority of the genes affected by nicotinamide at 24 h (Table 4). However, there was not a continued effect of nicotinamide treatment on the expression of the genes at 72 h although nicotinamide concentrations were maintained until time of sacrifice.

By 72 h post-TBI, 57 unique genes were significantly over-represented in the IPA identified significant pathways and categories in the NAM/vehicle contrast (Table 5). IPA analysis identified a significant effect of nicotinamide on cell-to-cell-signaling and interactions, molecular transport, small molecular biochemistry, cell, and drug metabolism. All of the top 10 canonical pathways identified were signaling pathways. In contrast to the effects at 24 h, the majority of the genes differentially expressed were up-regulated by nicotinamide in the NAM/vehicle contrast, were down-regulated by TBI. The genes differentially expressed with nicotinamide at 72 h were not affected by nicotinamide treatment at the earlier 24 h time point (Table 5).

### Validation of microarray data

#### Validation of microarray data

To validate gene expression changes, 15 genes were selected from specific pathways of interest. Figure 6 shows the gene expression results generated via microarray and qPCR (normalized to β-actin) for the nicotinamide versus vehicle group contrasts; Figures 6A,B show the data for the 24 and 72-h time points respectively. The qPCR findings were highly correlated with the microarray data (Slope = 1.187, Pearson’s *R* = 0.54).

## Discussion

In a study comparing a nicotinamide infusion to two doses of progesterone on functional recovery in the CCI model, the neuroprotective effect of nicotinamide, was equally effective asa low dose progesterone (10 mg/kg i.p. every 12 h for 72 h; Peterson et al., 2012). The functional recovery study used the same experimental conditions (rat strain, injury model, nicotinamide dosing regimen, and nicotinamide dose) as this study used. In fact, the samples that were used in this study were generated by the same laboratory. Therefore, the phenotypic data described by Peterson and colleagues can be directly compared to the transcriptional changes described in this study.

Both the nicotinamide infusion and the low dose progesterone were significantly more effective than the higher dose progesterone (20 mg/kg i.p. every 12 h for 72 h; Peterson et al., 2012). Functional recovery was assessed with two spatial memory tasks in the Morris water maze, the acquisition of a reference memory task and a reversal learning task. Neuropathological assessments were conducted in the cortex and hippocampus. Both low dose progesterone and nicotinamide improved reference memory acquisition and reversal learning in the Morris water maze and reduced tissue loss in the injured cortex and ipsilateral hippocampus compared to vehicle treatment. As determined in this study, the mechanism of the effect of nicotinamide on secondary injury pathways involves effects on inflammatory response, signaling pathways, and cell death.

### Inflammatory response

At 24 h, nicotinamide down-regulated the expression of 23 genes involved in immune or inflammatory processes. All 23 were significantly up-regulated in the TBI animals compared to sham controls and included cytokines, cytokine receptors, and chemokines as well as others involved in the inflammatory/immune response. Nicotinamide treatment down-regulated the expression of Il-1β, Il-receptor type II (Ilr2), Il-1 receptor antagonist (Il1rn), Il-8 receptor β (Il-8rβ) tumor necrosis factor receptor superfamily, member 1β (Tnfrsf1β), and other genes involved in the inflammatory response (Tables 2 and 4). Nicotinamide treatment significantly altered the expression of genes involved in Il-10, Trem1, Il-6, Nf-κB. LXR/RXR, PPAR, NF-κB, and glucocorticoid signaling pathways at 24 h post-TBI (see Table 2 for specific genes involved in these pathways). Il-10 is the principal anti-inflammatory cytokine and inhibits the expression of the pro-inflammatory mediators, TNFα, Il-6, Il-1, Mmp9, and the generation of Nos2 (Murray and Smale, 2012). Interesting, neither TBI nor nicotinamide treatment changed expression of the Il-6 or Il-10 genes themselves at the time points evaluated. The expression of Trem1 was increased by TBI and decreased by nicotinamide treatment (Table 4). Trem1 increases the secretion of pro-inflammatory cytokines and chemokines (Colonna, 2003). Nicotinamide decreased the expression of c-type lectin domain family 4, member e (Clec4e), c-type lectin domain family 4, member d (Clec4d), and S100 calcium binding protein A9 (S100a9). In the CCI injured animals compared to sham control, TBI increased the expression of Clec4d and Clec4e, 6- and 10-fold respectively. Clec4d and Clec4e are induced by inflammatory stimuli and expression leads to cytokine and chemokine production (Yamasaki et al., 2008; Wong et al., 2011). S100A9 was increased 15-fold by TBI and regulates vascular inflammation by promoting leukocyte recruitment (Bourke et al., 2003; Croce et al., 2009).

Chemokines are regulatory peptides whose major role is to guide the migration and activation of leukocyte cells into inflammatory sites and are considered pro-inflammatory. Chemokines act with chemokine receptors, G-protein-linked transmembrane receptors found on surface of target cells. Chemokine ligand 2 (Ccl2) and its receptor chemokine receptor 2 (Ccr2) are both increased in traumatic and ischemic injuries and have been implicated in their neuropathologies (Semple et al., 2010c). TBI significantly increased the gene expression of several chemokines and chemokine receptors including a 44-fold increase in Ccl2 (Table 5) and 3-fold increased expression in its receptor Ccr2. In a knock-out mouse model of closed head injury, Ccl2−/− mice had smaller cortical lesions and decreased neuronal loss (Semple et al., 2010a,b). Treatment with nicotinamide significantly down-regulated the gene expression of Ccl3, Ccl6, Ccl7, Cxc12, and colony stimulating factor 3 receptor (Csf3r). Nicotinamide did not decrease the expression of either Ccl2 or Ccr2.

LXR, PPAR, and FXR are nuclear receptors which when activated, translocate into the nucleus, form a dimer, or heterodimer with RXR to up or down regulate gene expression. Nicotinamide decrease the gene expression of only one nuclear receptor, Nfkbia (Table 4). Nfkbia, is a member of the NF-κ-B inhibitor family, which are involved in the expression of the pro-inflammatory cytokines, immune response, and anti-apoptotic genes (Tak and Firestein, 2001). Polymorphisms of the Nfkbia gene are associated with susceptibility to autoimmune and inflammatory diseases (Zhang et al., 2011).

At 72 h, post-TBI, nicotinamide increased the expression of prostaglandin-endoperoxide synthase 2 (Ptgs2), also known as COX-2 (Table 5). Ptgs2 can be either pro-inflammatory or anti-inflammatory (Choi et al., 2009). Ptgs2 is localized in neurons and is mainly induced in response to inflammatory stimuli. Animal studies have shown that during the early phase of inflammation, Ptgs2 is pro-inflammatory, but during the later phases of inflammation dominated by mononuclear cells, Ptgs2 appears to have anti-inflammatory effects by generating an alternate set of anti-inflammatory prostaglandins (Gilroy et al., 1999). Nicotinamide treatment resulted in increased gene expression of the RT1 and RTII class proteins, the major histocompatibility complex (MHC) of the rat (Gunther and Walter, 2001). In addition to their function in adaptive immunity, MHC class 1 molecules have been shown to be important in brain development, neuronal differentiation, and synaptic plasticity (Boulanger and Shatz, 2004).

### Signaling pathways

At 72 h, nicotinamide increased the expression of several hormones and receptors including the expression of two subunits of the GABA_A_ receptor, GABA_A_ receptor epsilon and GABA_A_ receptor, α3 (Table 5). The GABA_A_ receptor is a ligand-gated chloride channel, which binds GABA, the major inhibitory neurotransmitter in the brain. Nicotinamide also increased the gene expression of the glutamate receptor, ionotropic, kainate 3 (Grik3), serotonin receptors 1F and 2A, adrenergic α-1B, receptor, histamine receptor H3, relaxin/insulin-like family peptic receptor 2, and the somatostatin receptor 1 (Table 5). In the brain, the histamine receptor H3, is an auto-receptor, inhibiting the release of histamine (Schwartz, 2011). Nicotinamide increased the expression of somatostatin and its receptor. Somatostatin is a growth hormone-inhibition hormone, responsible for inhibiting the release of numerous secondary hormones. Somatostatin has been shown to have anticonvulsant activity in models of status epilepticus (Vezzani et al., 1991; Tallent and Qiu, 2008).

Nicotinamide increased the gene expression of several potassium and sodium voltage-gated ion channels, as well as, calcium voltage-gated ion channels. Specifically, up-regulation of potassium channels has been associated with diminished neural excitability. Potassium channel modulators have been proposed as neuroprotective drugs (Leung, 2010). The expression of synaptic vesicle glycoprotein 2b (Sv2b) was decreased with TBI and increased by nicotinamide. Synaptic vesicle proteins have been implicated with the etiology of epilepsy (Janz et al., 1999; Feng et al., 2009). SV2A has been identified as a target of the antiepileptic drug, levetiracetam (De Smedt et al., 2007), and has recently been found in patients with temporal lobe epilepsy (Janz et al., 1999; Feng et al., 2009). SV2B has been shown to regulate presynaptic Ca^2+^ signaling and has also been suggested as a target for treatment of epilepsy (Wan et al., 2010).

### Apoptosis

The functional category “cell death” was among the top 10 categories with genes significantly over-represented at the 24-h time point (Nam/vehicle; Figure 3). Nicotinamide is a low potency endogenous inhibitor of PARP (Cantoni et al., 1987; Virag and Szabo, 2002). PARP is an enzyme found in the nuclei of cells and functions to sense DNA damage, binds to the damaged DNA, catalyzes the cleavage of NAD+ into nicotinamide and ADP-ribose, and then uses the ADP-ribose to synthesize polymers which attach to nuclear acceptor proteins (Virag and Szabo, 2002). In addition, to the effect of PARP on DNA repair and apoptosis, PARP enhances NF-(B-mediated transcription, and regulates the formation of inflammatory mediators of inducible nitric oxide synthase (Nos2). Nitric oxide is a major signaling molecule in the nervous, immune, and circulatory systems with its action restricted to local effects due to its instability. Nos2 was up-regulated following TBI compared to sham controls at 24 h after injury and nicotinamide decreased the gene expression (Table 4). In the present study there were no effects of TBI or nicotinamide treatment on the expression of PARP1, the major isoform present in the nucleus.

## Conclusion

The proposed mechanism of the beneficial effect of nicotinamide (anti-inflammatory, anti-oxidant, and preventing apoptosis) has been based on experimental studies with 10–30 times higher concentrations than used in this study (Yang and Adams, 2004; Li et al., 2006). In this study, we demonstrated that at lower concentrations, the primary effect of nicotinamide is on inflammatory pathways at 24 h post-TBI. By 72 h post-TBI, the primary effect of nicotinamide is on cell signaling pathways involving neurotransmitters, neuropeptides, growth factors, and ion channels with little to no effect on inflammatory pathways.

Another potential promising treatment for TBI is progesterone which has been studied in preclinical models of TBI for several decades (Stein and Wright, 2010) and there are currently two large multi-center clinical TBI studies on-going. In a study evaluating the effect of the progesterone dose on gene expression in the same CCI injury model (Anderson et al., 2011), there was a significant effect of progesterone on inflammatory responses, apoptosis, regulating the DNA damage response, cell proliferation, and blood vessel remodeling following TBI. Low dose progesterone treatment significantly altered the expression of 14 and 40 genes at 24 and 72 h, respectively with approximately equal number up- or down-regulated. At 7 days, low dose progesterone up-regulated 383 of 551 (70%) genes compared to vehicle compared to the lack of effect of nicotinamide on gene expression at 7 days. In general, nicotinamide treatment counteracted the changes in genes differentially expressed due to TBI, while progesterone treatment primarily altered expression of genes that were not affected directly by TBI itself.

In conclusion, the mechanism of the effect of nicotinamide on secondary injury pathways involves effects on inflammatory response, signaling pathways, and cell death. The unique effect of nicotinamide compared to progesterone on gene expression suggests that a combination of nicotinamide and low dose progesterone may be an effective combination of treatment for TBI.

## Conflict of Interest Statement

The authors declare that the research was conducted in the absence of any commercial or financial relationships that could be construed as a potential conflict of interest.

## Footnotes

^1^www.myneurolab.com

^2^www.ingenuity.com
